# Extensive Drug Resistant Acinetobacter Species Isolates in Sputum Sample of Patient Admitted in Intensive Care Unit of a Tertiary Care Centre: A Descriptive Cross-sectional Study

**DOI:** 10.31729/jnma.7088

**Published:** 2021-10-31

**Authors:** Astha Prasai, Abhishek Pant, Asmita Neupane, Subhash Pant, Sailesh Pradhan

**Affiliations:** 1 Department of Medicine, HAMS Hospital, Dhumbarahi, Kathmandu, Nepal; 2 Department of Medicine, Grande International Hospital, Dhapasi, Kathmandu, Nepal; 3 Kathmandu Medical College and Teaching Hospital, Sinamangal, Kathmandu, Nepal; 4 Department of Medicine, Kathmandu Medical College and Teaching Hospital, Sinamangal, Kathmandu, Nepal; 5 Department of Pathology, Kathmandu Medical College and Teaching Hospital, Sinamangal, Kathmandu, Nepal

**Keywords:** *acinetobacter*, *antibiotics*, *extensive*, *resistance*

## Abstract

**Introduction:**

Increasing antibiotic resistance has created a global public health threat worldwide. Acinetobacter species is one of the important pathogenic organisms in the hospital setting due to its ability to persist in the hospital environment for long. Its resistance to commonly used antibiotics can prolong hospital stay, increase financial burden, and increase morbidity and mortality. This study aims to find the prevalence of extensive drug resistant Acinetobacter species in the sputum sample of Intensive Care Unit patients admitted in a tertiary care center.

**Methods:**

A descriptive cross-sectional study was conducted in a tertiary care center among the hospital records from May 2017 to May 2021, after ethical approval (Reference number: 2104202101). Hospital records of all Intensive Care Unit patients with Acinetobacter species isolated in their sputum sample within the past four years were collected and Statistical Package for Social Sciences version 25 was utilized for analysis. Point estimate at 95% Confidence Interval was calculated along with frequency and proportion for binary data.

**Results:**

Of the total 409, 196 (47.9%) (95% Confidence Interval= 43.06-52.74) of Acinetobacter species in the sputum sample had extensive drug resistance. Of these, 193 (98.5%) and 1 (0.5%) of the extensive drug resistant Acinetobacter species were resistant to carbapenem and polymyxin respectively.

**Conclusions:**

Prevalence of extensive drug resistant acinetobacter was found higher compared to other studies.

## INTRODUCTION

Antimicrobial resistance has been declared by the WHO to be among its top ten global public health burden. At least 700,000 people die each year due to drug resistant diseases and if the current trend continues, ten million deaths could occur by 2050.^12^ Among the ESKAPE bugs, Acinetobacter species resistance has become one of the major concerns due to its ability to resist desiccation, accumulate in the environmental surfaces for prolonged period and its complex epidemiology.^3,4^

Acinetobacter species showed susceptibility to commonly used antibiotics like chloramphenicol, ampicillin, gentamicin and nalidixic acid during 1970s. In late 1970s, it became one of the significant nosocomial pathogens demanding the use of broad-spectrum antibiotics.^5,6^ Multidrug resistant Acinetobacter species is on rise since the past few decades.

This study aims to find the prevalence of extensive drug resistant Acinetobacter species in the sputum sample of Intensive Care Unit (ICU) patients admitted in a tertiary care center.

## METHODS

This descriptive cross-sectional study was carried out in Kathmandu Medical College and Teaching Hospital. Ethical approval was obtained from the IRC of Kathmandu Medical College and Teaching Hospital (Ref: 2104202101). All the samples in the hospital records available over the past four years were included in our study. We collected all samples from hospital records of ICU patients within the past 4 years from May 2017 to May 2021. All ICU patients who had Acinetobacter species isolated in their sputum sample were included in our study. ICU patients with other pathogens in their sputum sample and non-ICU patients with Acinetobacter species isolated in their sputum sample were excluded from the study. Sample size calculation was done as follows:

n = Z^2^ × p × q / e^2^

  = (1.96)^2^ × 0.5 × 0.5 / (0.05)^2^

  = 0.9604 / 0.0025

  = 385

where,

n = sample size
Z = 1.96, at 95% confidence interval
p = prevalence taken as 50%
q = 1-p
e = margin of error, 5%

However, a total of 409 sputum samples were included in the study. Information on Antibiotic susceptibility detected by Kirby Bauer Disc Diffusion method was used and entered in Statistical Package for Social Sciences version 25 and analysis was done. Point estimate at 95% confidence interval and frequency and proportion was calculated for binary data.

## RESULTS

Out of 409, 196 (47.9%) (95% Confidence Interval = 43.06-52.74) of ICU patients with Acinetobacter species in their sputum sample had extensive drug resistance.

**Table 1:** Extensive drug resistant Acinetobacter species showing resistance to different classes of drugs (n = 196).

Drug	n (%)
Extended spectrum cephalosporin	195 (99.5)
Penicillin + beta lactamase inhibitor	195 (99.5)
Antipseudomonal penicillin + beta lactamase inhibitor	196 (100)
Folate pathway inhibitor	195 (99.5)
Fluoroquinolone	194 (98.9)
Aminoglycoside	193 (98.5)
Tetracycline	196 (100)
Carbapenem	193 (98.5)
Polymyxin	1 (0.5)
Tigecycline	0 (0)

The sputum sample was not subjected to all the antibiotics in a particular drug class, but to at least a single agent of a class of drug mentioned above.

**Table 2:** Extensive drug resistant Acinetobacter species in different age groups (n= 196).

Age range (in yrs)	n (%)
0-20	8 (4.1)
20-40	41 (20.9)
40-60	51 (26.1)
60-80	84 (42.8)
80-100	12 (6.1)

## DISCUSSION

Major risk factors for the development of extensive drug resistance among Acinetobacter species include invasive procedures, indwelling catheters, prolonged hospital stay, mechanical ventilation and use of broad-spectrum antibiotics.^7-9^ Since the identification of Acinetobacter as a cause of nosocomial infection in early 1970s, multiple classes of antibiotics including beta lactams, sulphonamides, macrolides, aminoglycosides, fluoroquinolones, carbapenems, colistin and tigecyclines have been introduced at different point of time for the control of infection.^5,6,9^ However, antibiotic resistance is ever increasing. In that respect, sensitivity pattern to extended spectrum cephalosporin, penicillin + beta lactamase inhibitors, antipseudomonal penicillin+beta lactamase inhibitors, aminoglycosides, antipseudomonal fluoroquinolones, antipseudomonal carbapenems, folate pathway inhibitors, polymyxins and tetracyclines are used to define multidrug resistance (MDR), extensive drug resistance (XDR) and pan drug resistance (PDR). MDR is defined as non-susceptibility to ≥1 agent in ≥3 antimicrobial categories. XDR is defined as nonsusceptibility to ≥1 agent in all but ≤2 categories. PDR is defined as non-susceptibility to all antimicrobial agents.^10^

In our study, among 409 sputum samples, all were Acinetobacter baumannii and 47.9% were extensively drug resistant to clinically relevant antibiotics except colistin and tigecycline. In a study conducted in a tertiary hospital in Lahore, Pakistan from September 2020 to December 2020, among 174 A. baumannii isolates, 64.9% isolates were resistant to carbapenem (CR-AB) and all of these were extensively resistant to existing antibiotics, except colistin.^11^ The percentage of extensive drug resistant isolates in our study is higher compared to a study conducted in Saudi Arabia from October 2014 to January 2015, where 36% of the A. baumannii isolates were extensively resistant to tested antibiotics and a study in China where 17.87% of the isolates were extensively drug resistant.^12,13^ In the context of Nepal, in a study conducted in a tertiary hospital from December 2013 to December 2014, nearly half of the isolates were resistant to majority of antibiotics except polymyxin and tigecycline.^14^ In another study conducted in Nepal by Sapkota, et al. from July 2018 to January 2019, out of 384 isolates, most were resistant to commonly used antibiotics.^15^

In our study, among the XDR isolates, resistance to the extended spectrum cephalosporin, penicillin + beta lactamase inhibitor and antipseudomonal penicillin + beta lactamase inhibitor was 99.5%, 99.5% and 100% respectively. Furthermore, resistance to folate pathway inhibitor, fluoroquinolone, aminoglycoside, and tetracycline was 99.5%, 98.9%, 98.5% and 100% respectively. Our study showed higher resistance to antipseudomonal penicillin + beta lactamase inhibitor compared to a study performed by Sapkota et.al in Kathmandu Medical College.^15^ In a study by Parajuli et al. in Tribhuvan University Teaching Hospital, resistance to commonly used drugs was high, almost similar to our study.^16^ In our study, carbapenem resistance was 98.5% among the XDR isolates. In the study by Parajuli et al, of the total 51 isolates, around 86% and 84% of the isolates were resistant to imipenem and meropenem respectively. In another study by Joshi et al, among 44 isolates, 97.7% were resistant to carbapenem.^16,17^

In our study, 84 (42.8%) of the XDR isolates were found in the 60-80 years of age group and 8 (4.1%) were found in the 0-20 years of age group. In a study by Raut et al in Universal College of Medical Sciences, among 105 patients, age group 16-45 years was the one most infected by A. baumannii, though the percentage of XDR was not analyzed.^18^ Of the total XDR isolates, in our study 73 (37.2%) were detected in the year 2017-2018 and the number reduced to 37 (18.9%) in the year 2020-2021. Though a definite conclusion cannot be drawn on decreasing trend, but the reduction in number of isolates is an encouraging finding. Increasing trend of resistance was seen in an antimicrobial surveillance program that was conducted in Latin America and in another study conducted in China.^19^,^20^

Antibiotics like polymyxin and tigecycline have become our last resort in the management of Acinetobacter species infection with increasing resistance to other clinically relevant and commonly used antibiotics. In our study, polymyxin resistance was found among 1 (0.5%) XDR isolates and none of the isolates were resistant to tigecycline. This shows that we have already started to see resistance to the ultimate antibiotic options we have till date. Cautious use of antibiotics to prevent the development of resistant strains along with infection control measures and contact precautions to prevent the spread of resistant strains need to be encouraged and further strengthened to prevent further worsening of the scenario.

The present study has certain limitations. Measurement bias is a possibility in our study. The study has been conducted in only one institution and therefore may not be representative of the entire population. Extensive drug resistant Acinetobacter species has only been studied in our study. Similar study to evaluate the resistance of other pathogens will be of higher value. It is a descriptive study and analysis of different factors that may have contributed to the observed resistance has not been done in our study.

## CONCLUSIONS

Extensive drug resistant Acinetobacter species is found to be higher in our study compared to other studies done in similar settings. This information on the extensive drug resistant Acinetobacter species in the sputum sample of ICU patients in Kathmandu Medical College will be valuable for further strengthening antibiotic stewardship program. Judicious use of antibiotics and efforts to halt the spread of resistant strain through early detection of resistant strains and infection control measures should be encouraged.
